# Type B insulin resistance with glycemic extremes: a case report and literature review

**DOI:** 10.3389/fendo.2026.1852784

**Published:** 2026-05-22

**Authors:** Emmeline Monique T. Ngo, Jordan M. Rowe, Thilo Samson Chillon, Lutz Schomburg, Rebecca J Brown, Shourya Tadisina

**Affiliations:** 1 University of Missouri-Kansas City School of Medicine, Kansas, MO, United States; 2 University of Missouri-Kansas City School of Pharmacy, Kansas, MO, United States; 3 Department of Clinical Biochemistry, Odense University Hospital, Odense, Denmark; 4 Institute for Experimental Endocrinology, Charité-Universitätsmedizin Berlin, Berlin, Germany; 5 National Institute of Diabetes and Digestive and Kidney Diseases, National Institutes of Health, Bethesda, MD, United States

**Keywords:** anti-insulin receptor autoantibody, GLP-1 receptor agonists, insulin receptor antibody, rituximab, severe insulin resistance, Type B insulin resistance

## Abstract

**Background:**

Type B insulin resistance (TBIR) is a rare autoimmune disorder caused by immunoglobulin G (IgG) autoantibodies targeting the insulin receptor. These antibodies act as partial agonists, resulting in severe insulin resistance and hyperglycemia at high titer, and hypoglycemia at low titer. TBIR typically affects middle-aged women with autoimmune diseases but may occur in younger individuals and across diverse ethnic groups. Early recognition and timely immunosuppressive therapy are crucial for remission and prevention of metabolic complications.

**Case:**

We report a 20-year-old Hispanic woman who presented with diabetic ketoacidosis (DKA), severe refractory hyperglycemia, and extreme insulin resistance requiring high doses of U-500 insulin. She had cachexia, acanthosis nigricans, and persistent hyperglycemia despite intravenous insulin infusion exceeding 4 units/kg/day. A detailed clinical evaluation, biochemical testing, and immunologic studies were conducted to establish the diagnosis. Laboratory findings showed elevated C-peptide and adiponectin, with positive antinuclear and anti-Smith ribonucleoprotein antibodies. Insulin receptor antibody testing confirmed TBIR. The therapeutic approach included high-dose insulin therapy, adjunctive metabolic agents such as incretin agonists (GLP1 receptor agonist semaglutide), and immunosuppressive therapy, with continuous glucose monitoring (CGM) used for dynamic glucose assessment and titration. Adjunctive therapy with metformin and low-dose semaglutide reduced daily insulin requirements from over 5,000 units to 200 units. Subsequent treatment with rituximab and azathioprine led to complete discontinuation of insulin within one week. However, the course was complicated by serum sickness-like illness and recurrent fasting hypoglycemia, which was managed with glucocorticoids and diazoxide.

**Conclusion:**

This case illustrates a rare presentation of TBIR in a young Hispanic female, initially manifesting as refractory DKA and extreme insulin resistance, followed by antibody-induced hypoglycemia, highlighting the spectrum of TBIR. This biphasic glycemic course underscores the complex immunopathophysiology of TBIR. To our knowledge, this is the first reported case describing the use of a GLP-1 receptor agonist (semaglutide) to reduce insulin requirements in TBIR prior to immunosuppressive therapy. Continuous glucose monitoring proved essential in guiding therapy and preventing severe hypoglycemia. This case emphasizes the need for early diagnosis, individualized immunosuppressive regimens, and vigilant metabolic monitoring to optimize outcomes in patients with TBIR.

## Introduction

Severe insulin resistance syndromes are rare disorders characterized by marked hyperinsulinemia and profound impairment of insulin action, often leading to extreme hyperglycemia and metabolic complications. These syndromes are classified into genetic forms such as type A insulin resistance and acquired forms, most notably type B insulin resistance (TBIR) which is caused by autoantibodies targeting the insulin receptor (1–3).

TBIR is an acquired autoimmune disorder that predominantly affects middle-aged women (mean age ~44 years) and shows a high prevalence among African-American individuals (2, 4–6). It is strongly associated with other autoimmune diseases, most notably systemic lupus erythematosus, as well as rheumatoid arthritis and mixed connective tissue disease (2, 4, 5). Insulin receptor antibodies are immunoglobulin G (IgG) polyclonal antibodies directed against the insulin receptor. They exert a biphasic effect: autoantibodies initially behave as insulin-receptor agonists, stimulating glucose transport and oxidation despite simultaneously inhibiting insulin binding, but over subsequent hours their continued engagement of the receptor drives enhanced receptor internalization and degradation, resulting in progressive and profound insulin resistance (7, 8).

Most patients with TBIR present with refractory hyperglycemia despite massive insulin doses exceeding thousands of units per day (2–4, 9). Coexisting features often include weight loss, cachexia, acanthosis nigricans, and laboratory evidence of hyperinsulinemia along with detectable insulin receptor autoantibodies (1, 10, 11). Acanthosis nigricans is typically seen in the skin folds but can be observed in the periocular region, lips and other mucocutaneous tissues (12). Additional biochemical features of TBIR syndrome include elevated adiponectin levels and normal or low triglyceride levels despite severe insulin resistance (9, 12, 13). Of note, there are currently no universally accepted biochemical criteria for clinical diagnosis.

Although most patients present with severe insulin resistance and hyperglycemia, a subgroup of patients present with isolated hypoglycemia and suppressed insulin levels, reflecting intermittent receptor activation rather than blockade (2, 13, 14). Although hypoglycemia is an uncommon presenting feature of TBIR, many patients develop hypoglycemia during spontaneous or treatment-induced remission, when antibody titers are low (4, 12).

Early recognition is essential as timely immunosuppressive therapy can reverse severe metabolic derangements. A prospective clinical trial at the National Institutes of Health (NIH) demonstrated that combined immunosuppressive therapy achieved 86% remission rate with no mortality and a shorter treatment duration, suggesting TBIR may be a curable form of diabetes (6). A recent systematic review reported a mortality rate of around 15%, remission rate of 70% and a relapse rate of only 7% (4).

We present a case of TBIR, initially manifesting as refractory diabetic ketoacidosis (DKA) requiring exceptionally high insulin doses. The patient responded favorably to immunosuppressive therapy and subsequently developed recurrent hypoglycemia, underscoring the dynamic and challenging glycemic course characteristic of TBIR.

## Case presentation

**Figure 1:**
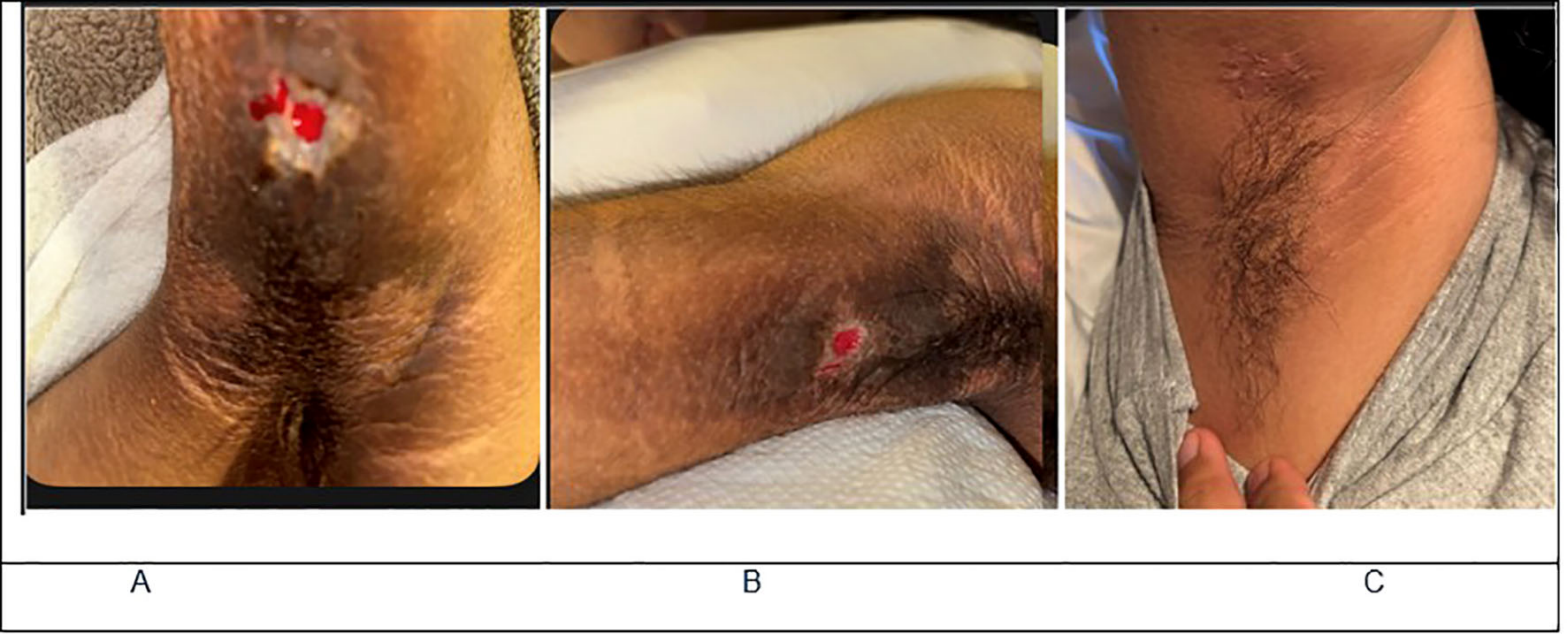
**(A, B)** show acanthosis nigricans in our patient prior to treatment. **(C)** Shows complete resolution of acanthosis nigricans after treatment.

A previously healthy 20-year-old Hispanic woman with a recent diagnosis of diabetes mellitus presented with severe, refractory, diabetic ketoacidosis (DKA), precipitated by a breast abscess which was appropriately managed with incision and drainage and antibiotics. Despite adequate treatment there was no response to standard DKA management, instead requiring >50 units/hour of intravenous insulin along with insulin glargine 100 units twice daily. Her history was notable for rapid 40-lb weight loss, recurrent DKA episodes, secondary amenorrhea, acne, hidradenitis suppurativa with recurrent infections, and marked acanthosis nigricans (**Figures 1A, B**). After ten days of treatment her DKA resolved and she remained profoundly hyperglycemic with insulin requirements exceeding 4 units/kg/day.

Laboratory evaluation (**Table 1**) revealed negative antibody testing for type 1 diabetes mellitus, elevated C-peptide, adiponectin, and sex hormone binding globulin, with a normal lipid panel. Evaluation for secondary causes of insulin resistance (including hypercortisolism, acromegaly, and insulin-binding antibodies) was negative. The possibility of type A insulin resistance was considered, but this was deemed less likely due to the absence of a relevant family history and lack of neonatal or childhood hypo- or hyperglycemia. The laboratory values demonstrating elevated adiponectin level and normal lipid panel raised concern for TBIR. ANA and anti-SmRNP antibodies were present, but the patient did not meet diagnostic criteria for systemic lupus erythematosus (SLE) or other systemic autoimmune diseases. Genetic testing for type A insulin resistance was planned only if TBIR testing returned negative. Insulin receptor antibody testing [conducted as described by Manikas et al. (11)] was strongly positive.

**Table 1:** Pertinent lab values.

Laboratory test	Admission	Immediately prior to immune-suppression	Post immuno- suppression	Hypoglycemic phase	Normal range
Random BG, (mg/dL)	385	91 (fasting)	180	82	<140
BHB (mmol/L)	>7.4				<0.27
Bicarbonate (meq/L)	5		20	27	22-32
Anion gap (mmol/L)	21		9	10	8-16
Arterial pH	6.9				7.3-7.45
Hemoglobin A1c, (%)	9.2	8.5	7.9	5.2	<5.7
T1DM Antibodies (GAD, ZnT8, ICA)	Negative				
C-peptide (ng/mL)	6.24	5.2	3.4		0.80-3.85
Insulin (mcU/mL)		341.7	64		2.6-24.9
AST (U/L)	44	19	25	23	15-41
ALT (U/L)	32	15	38	19	14-54
ALP (U/L)	108	100	88	103	35-104
Total cholesterol (mg/dL)	189	151	114		0-200
Triglycerides (mg/dL)	118	79	111		10-150
HDL (mg/dL)	61	68	46		40-60
LDL (mg/dL)	104	69	48		65-175
Leptin (ng/mL)	0.7				Females 4.7-23.7
Adiponectin (ug/mL)	>40				Female BMI <25:2.9-30.4
SHBG (nmol/L)	226	141			17-124
ANA Screen	Positive	Positive			Negative
dS DNA	7	17	17		>10 positive; 5–9 indeterminate
SmRNP	3		8.0		<1 AI: negative >1 AI: positive
Complement C4 (mg/dL)	12.03	12			12.9-39.2 (15–57 for NIH lab)
C-Reactive protein (mg/L)	1.6	1.2			<10 (0–5 for NIH lab)
Insulin receptor antibody	Positive				
Antibody binding index	331				≤3.4*
CD19, mcL		392	13		61-321

T1DM, type1 diabetes mellitus,;BG, blood glucose; BHB, betahydroxybutyrate.

GAD, Glutamic acid decarboxylase; ZnT8, Zinc transporter 8; ICA, islet cell antibodies, islet antigen 2; AST, Aspartate aminotransferase; ALT, Alanine aminotransferase; ALP, Alkaline Phosphatase; HDL, high density lipoprotein; LDL, Low density lipoprotein; SHBG, Sex hormone binding globulin; ANA, antinuclear antibody; ds DNA, double stranded DNA; SmRNP, Smith Ribonucleoprotein.

* A binding index ≤3.4 has 91.3% sensitivity and 93.9% sensitivity to detect Type B insulin resistance, while a binding index >29.7 has 96.5% sensitivity and 100% specificity for severe Type B insulin resistance.

Given her extremely high insulin resistance, she was transitioned to concentrated Humulin R U-500 insulin and titrated to 5,100 units/day within four weeks. In addition to the challenge of optimally managing her insulin resistance, the logistics of obtaining sufficient insulin to meet the patient’s requirements was also complicated by initial lack of insurance coverage for U-500 insulin, necessitating cash pay. The authors’ institution is 340B-eligible and at the time of the patient’s management, the 340B cash price for U-500 products was under $30 for a 15-day supply, which the patient was able to utilize. She was initially prescribed U-500 pens for safety but had to quickly be transitioned to U-500 vials as each dose required multiple injections on the pen device (max dose of 300 units per injection) and one pen’s volume was not sufficient for even one of her multiple daily insulin administrations. She received individualized injection education following hospital discharge to ensure safe administration of U-500 insulin, and the clinical team coordinated with her community pharmacy to prevent delays in dispensing due to initial concerns for transcription errors with the over 5000 total daily insulin units prescribed.

**Figure 2:**
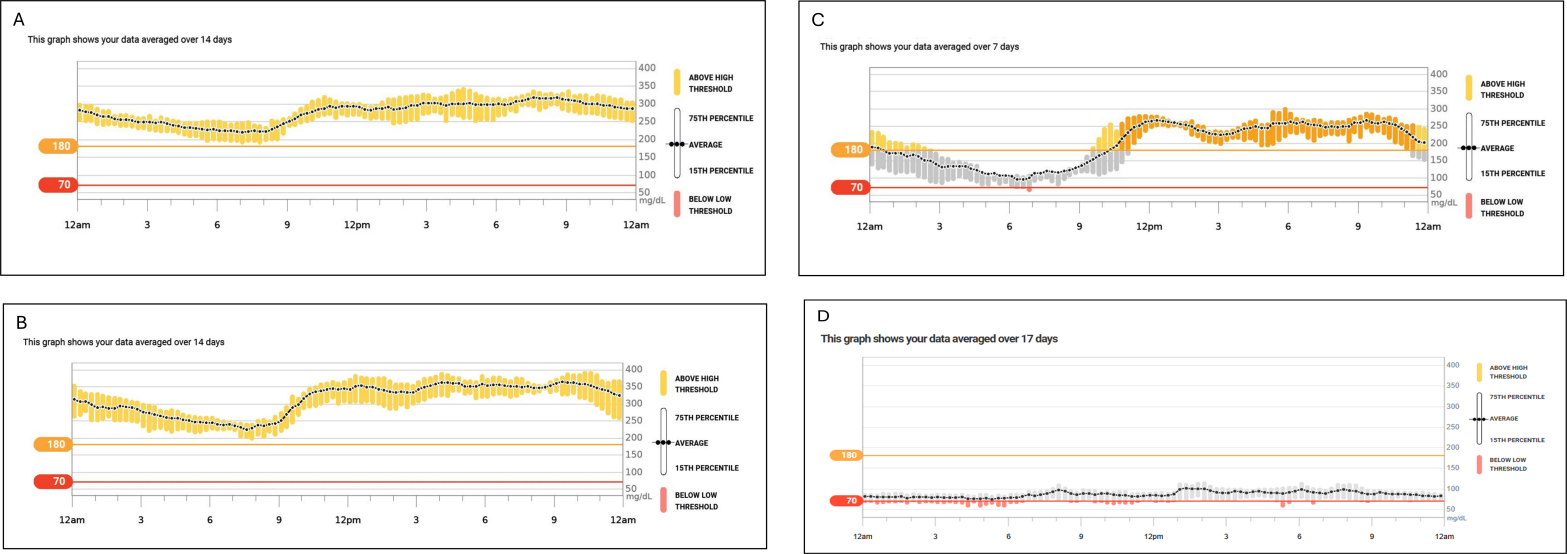
Continuous glucose monitor readings during insulin titration over a period of 6 weeks from hospital discharge; **(A)** At 2 weeks from discharge on Humulin R U-500–850 units/day; **(B)** At 4 weeks from discharge on Humulin R U-500–4500 units/day with semaglutide 0.25 mg/weekly and metformin 1000 mg twice daily; **(C)** At 6 weeks from discharge when she first noticed fasting hypoglycemia on Humulin R U-500–5100 units/day with semaglutide 0.5 mg/weekly and metformin 1000 mg twice daily. **(D)** Hypoglycemia after immunosuppression therapy and development of serum-like sickness, which necessitated pulsed glucocorticoid administration. These hypoglycemic episodes were initially responsive to high-dose glucocorticoids, with recurrence after taper and eventually required diazoxide for control.

As these substantial doses of insulin created considerable injection burden, a weekly glucagon-like peptide-1 (GLP1) receptor agonist (semaglutide) and metformin were initiated three weeks after discharge with close monitoring by continuous glucose monitor (CGM). Her insulin requirement decreased to 200 units by ten weeks. GLP-1 therapy was used for about 7 weeks (3 weeks post-hospital discharge to 1 week pre-immunosuppressive therapy). Her BMI increased from 18 (48 kg) to 21.3 (57.5 kg) with improved glycemic control on semaglutide and resolution of the hypercatabolic state. Nutritional status and protein intake were maintained. **Figure 2** displays the CGM data during the different stages of insulin titration.

**Figure 3:**
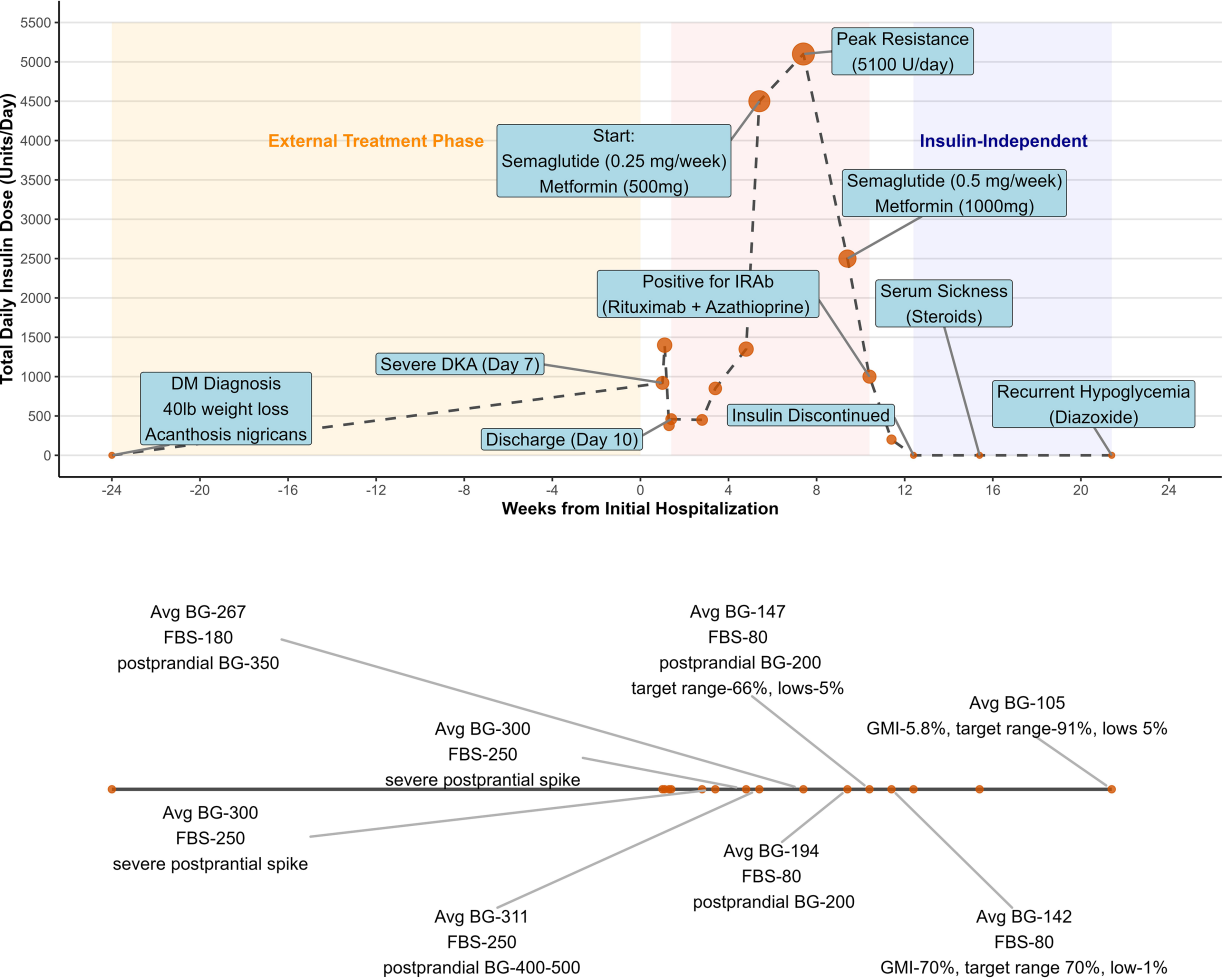
Longitudinal timeline of total daily insulin dose (TDD) and glycemic metrics relative to clinical events. The upper panel shows TDD (units/day) plotted against weeks from initial hospitalization, with key clinical events and therapeutic interventions annotated. Shaded areas indicate predefined clinical phases: External Treatment Phase (orange), period of Extreme Insulin Resistance (red), and Insulin-Independent Phase (blue). The under panel displays corresponding continuous glucose monitoring (CGM) summaries at selected time points, including average blood glucose (Avg BG), fasting blood glucose (FBS), postprandial glucose levels, glucose management indicator (GMI), time in range, and hypoglycemia percentage.

She subsequently received immunosuppressive therapy with rituximab and azathioprine, which enabled complete discontinuation of insulin and all antidiabetic medications within one week. Her course was complicated by a serum sickness-like illness following rituximab, which prevented administration of the second dose; however, her CD19 levels three weeks post-treatment were appropriately suppressed as shown in **Table 1**. Shortly thereafter, she developed recurrent hypoglycemia. Pulsed high-dose glucocorticoids administered for serum sickness-like illness controlled the hypoglycemia; however, recurrent hypoglycemic episodes occurred after steroid tapering (**Figure 2D**) and was eventually managed with low-dose diazoxide. Acanthosis nigricans resolved within two months after treatment (**Figure 1C**). She is now able to attend college with a lower disease burden and has been off all antidiabetic agents for about eight months. **Figure 3** displays a timeline of the events along with the varying insulin requirements at different stages.

## Discussion

We describe a young Hispanic woman presenting with refractory DKA, profound acanthosis nigricans, extreme insulin resistance, and confirmed insulin receptor antibodies, consistent with TBIR. While TBIR most frequently occurs in middle-aged African American women, cases in younger patients and other ethnic groups, including Asian and Hispanic populations, are increasingly recognized (2–5, 15). Ethnic and geographic variation with earlier onset in some groups points to potential genetic and environmental influence, but evidence is limited. Her initial course was dominated by refractory hyperglycemia despite massive insulin doses, a hallmark of TBIR. Similar extreme insulin requirements have been documented in both Western and Asian cohorts (2, 9, 12, 15).

TBIR is often accompanied by weight loss and dermatologic signs of insulin resistance such as acanthosis nigricans (4, 9, 12). The presence of ketosis has been reported in only 11% of cases in a recent systematic review (4). However, our patient experienced several episodes of DKA, likely precipitated by recurrent infections. A published case series describing the management challenges of DKA in patients with TBIR or pathogenic *INSR* gene variants highlights that these episodes are typically triggered by underlying infections and often necessitate massive insulin doses. In one case, intravenous insulin requirements reached as high as 50,000 units per day, with resolution of acidosis achieved only after the underlying infection was treated (16).

Expedited insurance coverage was critical for our patient’s access to U-500 insulin as management with traditional U-100 basal/bolus insulin strategies would have been unable to meet her initial extreme insulin needs. Following the course of this patient, Humulin U-500 insulin’s manufacturer, Lilly, announced that the company would be sunsetting production of U-500 vials and the product will subsequently be only available in 3 ml pens (17). Although instances of extreme insulin resistance such as the case presented here are rare, the loss of U-500 vials will make logistical management of future cases of extreme insulin resistance even more challenging.

Only two reported cases in literature have described the use of GLP1 receptor agonists in TBIR, and in both these case scenarios the GLP-1 agent was initiated only after immunosuppressive therapy (18, 19). Our patient demonstrated a favorable response to injectable semaglutide even when initiated prior to immunosuppressive therapy, resulting in substantial reduction in insulin requirements as the dose was gradually up-titrated. However, the degree and timing of this improvement raises questions regarding its underlying drivers.

A marked reduction in insulin requirements prior to starting immunosuppression raises the possibility of evolving spontaneous remission in Type B insulin resistance. Prior studies have shown that partial or complete spontaneous remission can occur independent of immunosuppression, likely reflecting changes in autoantibody titer, affinity, or function that either reduce receptor blockade or exert agonistic effects (12). Similarly, systematic reviews note that spontaneous improvement is well described and may precede or complicate interpretation of treatment response (2, 4).

The marked decline in insulin requirements in our patient (from >5,000 units/day to 200 units/day within ten weeks prior to immunosuppression) suggests that partial remission may have been underway. Semaglutide was initiated during this period and may have contributed to improved glycemic control, making it difficult to separate the effects of incretin therapy from underlying disease modulation. Although rapid discontinuation of insulin following immunosuppression is consistent with treatment response, the preceding improvement highlights the challenge of distinguishing therapeutic effect from spontaneous waning of pathogenic autoantibody activity. Declining insulin requirements prior to immunosuppression may therefore reflect a remission trajectory, with immunotherapy potentially accelerating an already improving disease course (2, 4, 12).

To our knowledge, the therapeutic use of GLP-1 receptor agonists in the management of TBIR prior to immunosuppressive therapy has not been previously reported. While fasting hypoglycemia is a recognized manifestation of TBIR and may theoretically be exacerbated by weekly GLP-1 therapy, in this case semaglutide was initiated at a low dose along with close monitoring by CGM given the inability to achieve glycemic control despite rapid increases in insulin doses.

She ultimately received immunosuppression rituximab, azathioprine, and pulsed high-dose glucocorticoids; this regimen was modeled after NIH protocols using rituximab, cyclophosphamide, and corticosteroids (6, 20). Unfortunately, our patient developed a serum sickness-like illness following therapy, precluding completion of the planned treatment course. While generally well tolerated, immunomodulatory regimens can be complicated by infections, infusion reactions, or immune-complex–mediated events (6, 15). Her transition from severe hyperglycemia to recurrent hypoglycemia that is responsive to diazoxide reflects the biphasic pattern described in TBIR. Insulin receptor antibodies in TBIR are polyclonal in nature, and different antibodies may target different epitopes of the receptor, yielding effects ranging from antagonism to partial agonism (8). Furthermore antibody effects may evolve from antagonistic to agonistic depending on the antibody titers, or insulin sensitivity may markedly increase during remission (11–13). This phenomenon underscores the need for vigilant glucose monitoring throughout treatment and recovery. The role of CGM in TBIR is not well established; however, given the biphasic pattern of the condition, CGM can facilitate close monitoring and insulin titration as demonstrated in a case reported with fasting hypoglycemia (14). The integration of CGM with insulin pumps has also been demonstrated in a TBIR patient following plasmapheresis and immunosuppressive therapy (21). In our case, CGM played a crucial role, allowing for real-time assessment of glycemic patterns and enabling rapid insulin adjustments to mitigate the risk of severe hypoglycemia.

Available literature supports that TBIR, although rare, is potentially reversible with prompt diagnosis and aggressive immunotherapy tailored to comorbid autoimmune conditions (4–6, 10, 21). Delays in diagnosis contribute to prolonged metabolic instability and increased morbidity, including risk of diabetic ketoacidosis, severe hypoglycemia, and complications of high-dose insulin therapy (5, 21, 22).

Management strategies can vary, as demonstrated by a case treated successfully with low-dose immunosuppressive therapy, indicating that less intensive treatment regimens combining corticosteroids and immunomodulatory agents may lead to gradual clinical improvement, decreased insulin requirements, and disappearance of insulin receptor autoantibodies. This suggests that low-dose regimens can be an effective alternative for patients who cannot tolerate aggressive therapy, advocating for tailored treatment plans based on patient tolerance and disease severity (20).

Our case adds to existing reports by illustrating TBIR in a young Hispanic woman without prior autoimmune diagnosis, complicated by an incomplete immunosuppressive course due to serum sickness-like illness, and subsequent hypoglycemia requiring diazoxide. This highlights both the therapeutic potential of targeted immunomodulation, as well as the need for individualized, adaptive management strategies in the setting of the unpredictable course of type B insulin resistance syndrome.

## Conclusion

This case highlights the diagnostic and therapeutic complexity of type B insulin resistance, particularly when it presents in an atypical demographic with life-threatening metabolic decompensation, leading to prolonged misdiagnosis. Our patient’s course illustrates the hallmark biphasic nature of TBIR, with an initial phase of extreme insulin resistance and refractory DKA requiring massive insulin doses, followed by treatment-associated remission complicated by recurrent hypoglycemia. The case underscores the importance of maintaining a high index of suspicion for TBIR in patients with severe insulin resistance, especially when also presenting with cachexia, acanthosis nigricans, preserved C-peptide, or autoimmune serologies, even in young individuals without a prior diagnosis of systemic autoimmune disease. CGM proved indispensable in navigating rapid transitions in insulin sensitivity and mitigating hypoglycemia risk during both escalation and de-escalation of therapy.

This report expands on currently published literature regarding the phenotypic spectrum of TBIR and documenting, to our knowledge, the first use of a GLP-1 receptor agonist as an adjunctive therapy to meaningfully reduce insulin requirements prior to definitive immunosuppression. It also highlights real-world logistical challenges in managing extreme insulin resistance, including access to concentrated insulin formulations, and emphasizes the need for individualized, adaptable treatment strategies in the context of variable treatment tolerance and unpredictable disease course. This case reinforces that TBIR, while rare and life-threatening without appropriate intervention, is a potentially reversible condition when recognized early and managed with collaborative approaches across disciplines.

## Patient perspective

“Getting a diagnosis was really, really difficult. I had the typical symptoms of a Type 1 diabetic and was often dismissed as being careless with the management of my diabetes. I had to be hospitalized five times for DKA before receiving a diagnosis. When I was finally diagnosed, I was relieved to find out that there was treatment available and that it would not be a life-long condition. I was not hesitant to receive treatment despite possible complications in the future. It was difficult to navigate the hypoglycemia at first, but it has become more manageable with medication and with my continuous glucose monitors. I’ve since regained my weight and have little trouble keeping my blood sugars in range”.

## Data Availability

The original contributions presented in the study are included in the article/supplementary material. Further inquiries can be directed to the corresponding author.
